# Functional epigenomic liquid biopsy identifies potential therapeutic vulnerabilities after enfortumab vedotin–pembrolizumab resistance in metastatic urothelial carcinoma (mUC)

**DOI:** 10.21203/rs.3.rs-11260686/v1

**Published:** 2026-10-19

**Authors:** Joaquim Bellmunt, Rong Li, Sara El Sarout, Yingtian Xie, Gunsagar Gulati, Ilana Epstein, Shweta Kukreja, Luisa Rodriguez, Stephanie Berg, Charlene Mantia, Bradley McGregor, Marina Kaymakcalan, Rashad Nawfal, Sylvan Baca, Toni Choueiri, Matthew Freedman, Henry Long, Paloma Cejas

## Abstract

Enfortumab vedotin plus pembrolizumab (EV/P) is the standard first-line treatment for advanced urothelial carcinoma (UC), yielding unprecedented clinical benefit; however, resistance ultimately develops in many patients, and longitudinal liquid biopsy offers an opportunity to monitor tumor evolution and emerging therapeutic vulnerabilities. We collected 96 serial plasma samples from 48 patients with advanced UC treated with first-line EV/P, of which 79 samples from 41 patients passed predefined quality-control criteria for H3K4me2 cfChIP-seq profiling. Progression during EV/P was accompanied by regulatory reprogramming characterized by recurrent increases in ERBB3-associated H3K4me2 activity together with DNA damage response, replication stress, hypoxia and metabolic adaptation programs. Longitudinal patient-level analyses identified heterogeneous increases in potentially therapeutically relevant loci, including ERBB2, FGFR3, TACSTD2, WEE1, CD276 and PPARG, that were not evident at baseline in selected patients. Among three platinum-treated patients with longitudinal samples, the two patients deriving clinical benefit showed declining ERCC2, ERBB3 and BRCA2 regulatory activity, whereas the patient with progressive disease showed increased ERCC2 and ERBB3 activity. In one illustrative patient, increased ERBB2 regulatory activity at EV/P progression preceded a durable response to trastuzumab deruxtecan. These findings support prospective evaluation of longitudinal functional epigenomic profiling as a complementary approach for characterizing resistance and informing biomarker-guided treatment sequencing after EV/P.

